# *sdrH* enhances *Staphylococcus aureus* infection in diabetic wounds

**DOI:** 10.3389/fmicb.2025.1502428

**Published:** 2025-06-19

**Authors:** Kaiyu Nie, Kaiyu Wang, Yin Wen, Jinmei Peng, Shijie Tang

**Affiliations:** ^1^Department of Plastic Surgery and Burn Center, Second Affiliated Hospital, Shantou University Medical College, Shantou, Guangdong, China; ^2^Plastic Surgery Institute of Shantou University Medical College, Guangzhou, China; ^3^Shantou Plastic Surgery Clinical Research Center, Guangzhou, China; ^4^Department of Burns and Plastic Surgery, Affiliated Hospital of Zunyi Medical University, Zunyi, China; ^5^The Collaborative Innovation Center of Tissue Damage Repair and Regeneration Medicine of Zunyi Medical University, Zunyi, China

**Keywords:** diabetes mellitus, diabetic ulcer, *Staphylococcus aureus*, infection, virulence factors

## Abstract

**Introduction:**

Diabetes mellitus is a widespread chronic condition that can lead to a variety of complications. Among the numerous complications associated with diabetes, diabetic foot ulcers are particularly notable due to their high prevalence and potential severity. These ulcers are characterized by a substantial incidence rate, a considerable risk of infection, and a high probability of necessitating amputation. *Staphylococcus aureus*, a notorious pathogen within this context, exacerbates wound pathogenesis and can facilitate ulcer extension and, in severe instances, gangrene through the secretion of numerous virulence factors.

**Methods:**

RT–qPCR was used to analyze the expression of *Staphylococcus aureus* adhesin virulence factors. Utilizing gene knockout techniques to deleted the *sdrH* and *icaA-C* genes. Biofilm formation of S. aureus was observed by scanning electron microscope. The effect of *sdrH* and *icaA-C* genes on *S. aureus* infected wound healing was also evaluated using a diabetic mice skin wound infection model.

**Results:**

The *sdrH* gene and the *icaA-C* gene cluster are critical contributors to *Staphylococcus aureus* infection in diabetic wounds. Post-infection with the *sdrH* single-gene knockout strain, a significant enhancement in wound healing rates was observed, accompanied by a marked reduction in bacterial colonization per unit area. Conversely, no significant differences were detected between the *icaA-C* gene cluster knockout strain and the wild-type strain. Compared to infections caused by either the wild-type strain or the *sdrH* single-knockout strain, infection with the *icaA-C*/*sdrH* double-knockout strain led to a marked increase in the wound healing rate and a significant reduction in bacterial load.

**Discussion:**

This study presented that the *sdrH* gene enhances the virulence of *Staphylococcus aureus* in diabetic wounds by attenuating the host immune response, reducing the infiltration of inflammatory cells, and impairing the immune system’s capacity to clear bacteria, thereby impeding the wound healing process. Although *icaA-C* is not a pivotal player, it seems to enhance the virulence capabilities of *sdrH*.

## 1 Introduction

Diabetic ulcers (DUs) represent significant chronic complications of diabetes, encompassing deep tissue lesions that are often linked to peripheral neuropathy and peripheral vascular disease, with diabetic foot ulcers (DFUs) being the most prevalent manifestation (Apelqvist, 2012). A global epidemiological survey published in 2017 reported that the average prevalence of DFUs among individuals with diabetes was 6.3% (95% confidence interval, 5.4–7.3%). This finding has heightened awareness of the issue among international medical promotion organizations, including the World Diabetes Foundation (WDF), and as well as various governments around the world (Sun et al., 2022). Despite the average prevalence of DFUs in China being lower than the global average, at 4.1% (95% confidence interval, 3.1–5.2%) (Zhang et al., 2017), the substantial diabetic population in China (approximately 129.8 million as of the latest epidemiological survey in 2020) (Li et al., 2020) renders the absolute number of patients with Dus or DFUs a significant public health concern. Foot ulcer infections are a prevalent, serious, and costly complication associated with diabetes. Diabetic ulcer infections (DUIs) predominantly arise from infections by diverse microorganisms. A recent meta-analysis encompassing 112 studies identified *Staphylococcus aureus* as the most prevalent pathogen in diabetic foot infections, with methicillin-resistant *Staphylococcus aureus* (MRSA) accounting for 18.0% of these cases, which cannot be ignored (Macdonald et al., 2021). The presence of bacteria in wounds can either directly or indirectly sustain inflammation, thereby hindering the healing process (Park et al., 2009; Schierle et al., 2009; Siddiqui and Bernstein, 2010; Fazli et al., 2011). The emergence of MRSA and vancomycin-intermediate *Staphylococcus aureus* (VISA) exacerbates the problem of bacterial resistance and may result in the failure of antibiotic treatments for staphylococcal infections (Hu et al., 2016). The interaction between wound-infecting bacteria and the host immune-inflammatory response is highly complex, and with bacterial factors playing crucial roles in determining clinical outcomes and the subsequent development of infections (David and Daum, 2010). Recent research has demonstrated that virulence factors constitute promising alternative targets for therapeutic intervention in *Staphylococcus aureus* infections (Mcadow et al., 2011; Chen et al., 2016). This bacterium is capable of producing wide array of virulence factors, including adhesins, toxins, and factors facilitating immune evasion (Zecconi and Scali, 2013). The pathogenic factors produced by *Staphylococcus aureus* exhibit strain specificity, yet the outcomes of infection can be attributed to the various combinations of pathogenic factors produced at diverse infection sites (Zecconi and Scali, 2013; Baba et al., 2008). For example, in a mouse model of skin abscess, the silencing of the immune evasion factor Staphylococcus A protein-encoding gene (spa), the fibronectin binding protein-encoding genes *fnbAB*, and *clfA* or surface protein-encoding gene *sasF*, resulted in a marked attenuation of the virulence of *Staphylococcus aureus.* However, the same modifications did not result in a significant alteration of pathogenicity in other infection models (Kwiecinski et al., 2014).

However, the key virulence factors involved in *Staphylococcus aureus* infections in DUs are not clear. In this study, we simulated high- and low-glucose growth environments *in vitro* and constructed a BALB/c mouse model of DU to replicate the infection process of the *Staphylococcus aureus* Newman strain in these lesions. The expression levels in 40 virulence determinants of *Staphylococcus aureus* Newman were analyzed via reverse transcription–real-time quantitative polymerase chain reaction (RT–qPCR) under different glucose concentration culture conditions in two groups, as well as in the BALB/c mouse DU model and a normal wound model. We found that high-glucose environments significantly upregulate the expression of bacterial extracellular polysaccharide-related genes (*icaA*, *icaD*, and *icaC*) and the adhesin gene *sdrH*. Therefore, we individual knock outs of the *sdrH* gene and the *icaA*-*C* gene cluster, and simultaneously knocked out both the *icaA*-*C* gene cluster and the sdrH gene from *Staphylococcus aureus* Newman, successfully generating Newman Δ*sdrH*, Newman Δ*icaA-C* and Newman Δ*icaA-C/*Δ*sdrH* strains. Subsequently, we utilized the knockout strains to infect DUs in a BALB/c mouse model and observed the wound healing process.

Our study identified critical virulence factors implicated in the pathogenesis of *Staphylococcus aureus* infection in DUs. These findings provide valuable insights that may inform the development of novel therapeutic targets aimed at mitigating virulence factors, thereby enhancing the treatment of *Staphylococcus aureus*-induced DUs.

## 2 Results

### 2.1 High sugar levels in the extracellular environment boost virulence factor expression in *Staphylococcus aureus*

**Figure 1:**
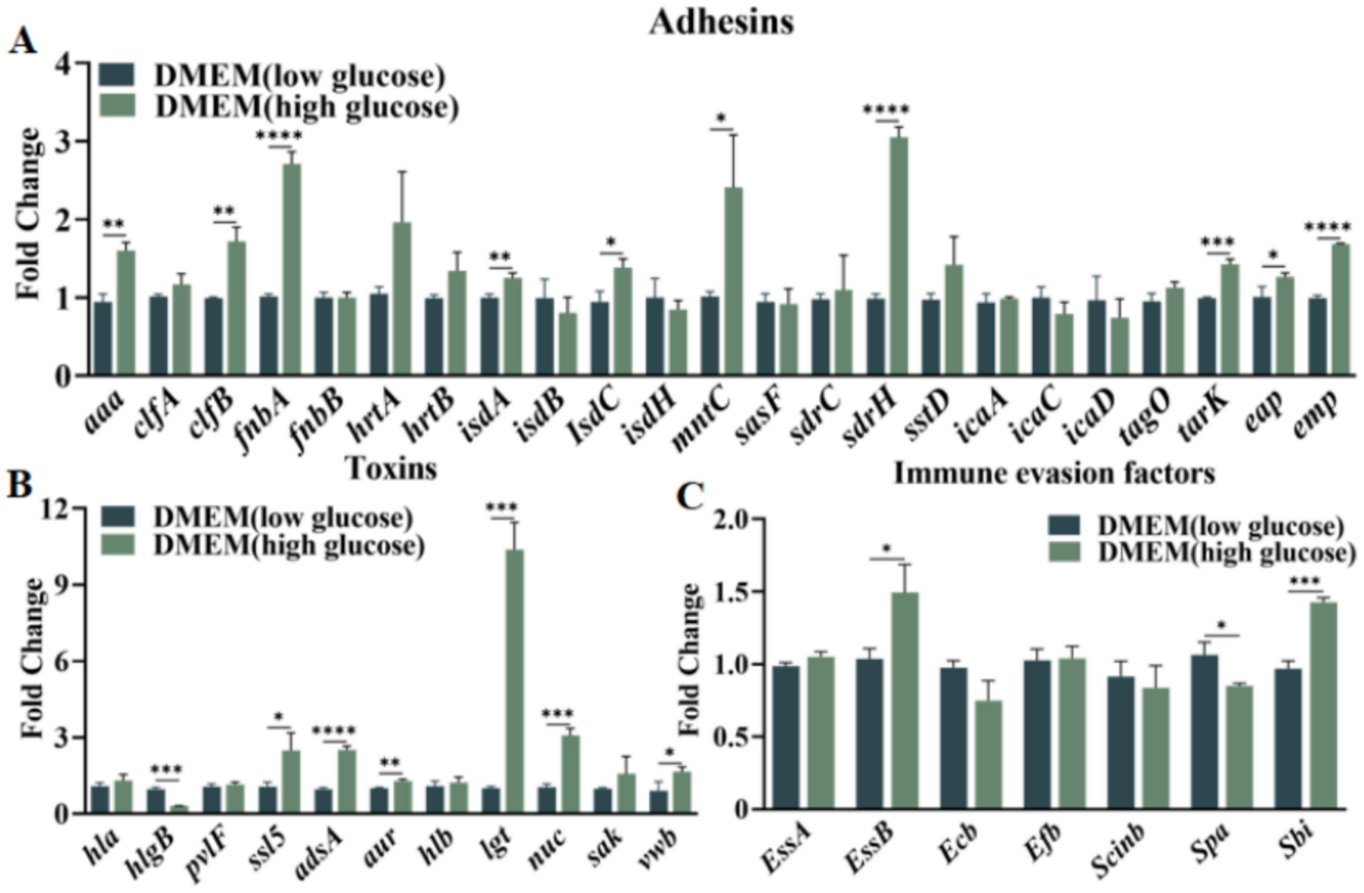
Panel **(A)** illustrates the expression of *Staphylococcus aureus* adhesin virulence factors in two DMEM-based media. Panel **(B)** demonstrates the expression of the *Staphylococcus aureus* toxins in two DMEM-based media. Panel **(C)** presents the expression of the *Staphylococcus aureus* immune evasion factors in two DMEM-based media. Statistical significance was determined using the t test, with * indicating *p* < 0.05, ** indicating *p* < 0.01, and *** indicating *p* < 0.001, and **** indicating *p* < 0.0001. The notation ‘ns’ denotes no statistical significance.

**Figure 2:**
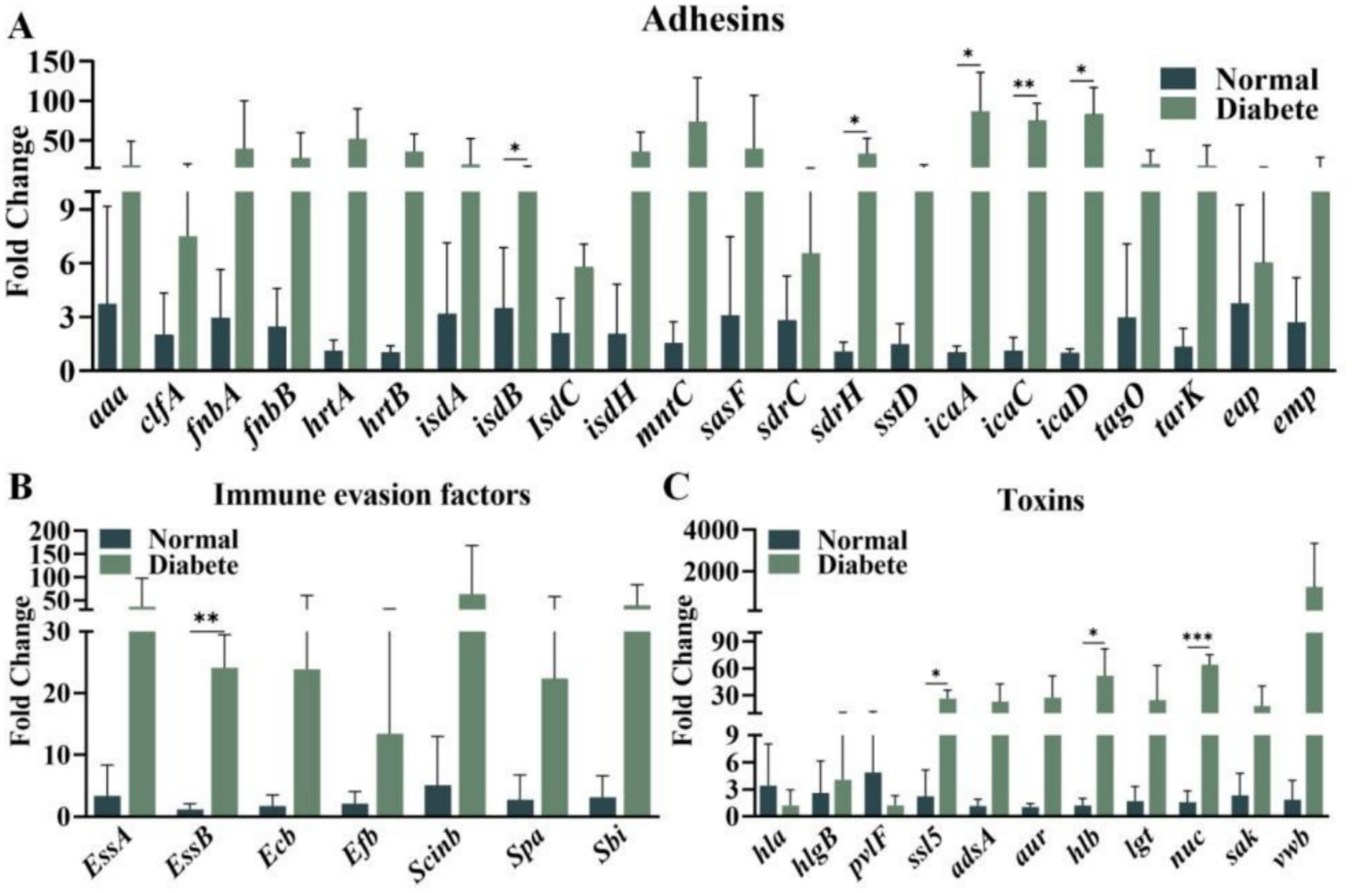
**(A)** The production of staphylococcal adhesin virulence factors in diabetic wounds versus non-diabetic wounds; **(B)** The expression of staphylococcal toxins in diabetic wounds versus non-diabetic wounds; **(C)** the expression of staphylococcal immune evasion factors in diabetic wounds compared to non-diabetic wounds were investigated. The *t* test was employed to ascertain statistical significance, with * indicating *p* < 0.05, ** indicating *p* < 0.01, and *** indicating *p* < 0.001; ns denotes no statistical significance.

*Staphylococcus aureus* virulence factors are predominantly categorized into adhesins, toxins, and immune evasion factors (Becker, 2018). The production of these virulence factors varies depending upon the environment. In diabetic wounds, hyperglycemia occurs and promotes bacterial colonization. A comparative analysis of the expression profiles of three types of virulence factors—adhesins, toxins, and immune evasion factors—in wounds from in mice exhibiting hyperglycemic and normoglycemic conditions was conducted *in vitro* by using medium with and without in a high-sugar, respectively.The results revealed a substantial increase in the expression levels of adhesin genes, including *aaa, clfB, fnbA, isdA, isdC, mntC, sdrH, tarK, eap,* and *emp*, in *S. aureus* subjected to an elevated glucose concentration (Figure 1A). Concurrently, the expression of toxin genes such as *hlgB, ssl5, adsA, aur, lgt,* and *nuc*, and the immune evasion factor *essb* also markedly upregulated (Figures 1B,C). With a mouse DFU model, *S. aureus* markedly upregulated the expression of some adhesin genes, including *isdB, sdrH, icaA, icaC,* and *icaD* (Figure 2A). A significant increase in the expression of toxin genes like *ssl5, hlb,* and *nuc*, and the immune evasion factor *essb* was also observed (Figures 2B,C). These findings demonstrated that a high-glucose environment induces the expression of various virulence factor genes such as *sdrH* and *icaA*-*C* in *S. aureus*. These upregulated genes may be subjected to manipulation of host immune response.

### 2.2 Genetic construction of the *sdrH*/*icaA*-*C* mutants

**Figure 3:**
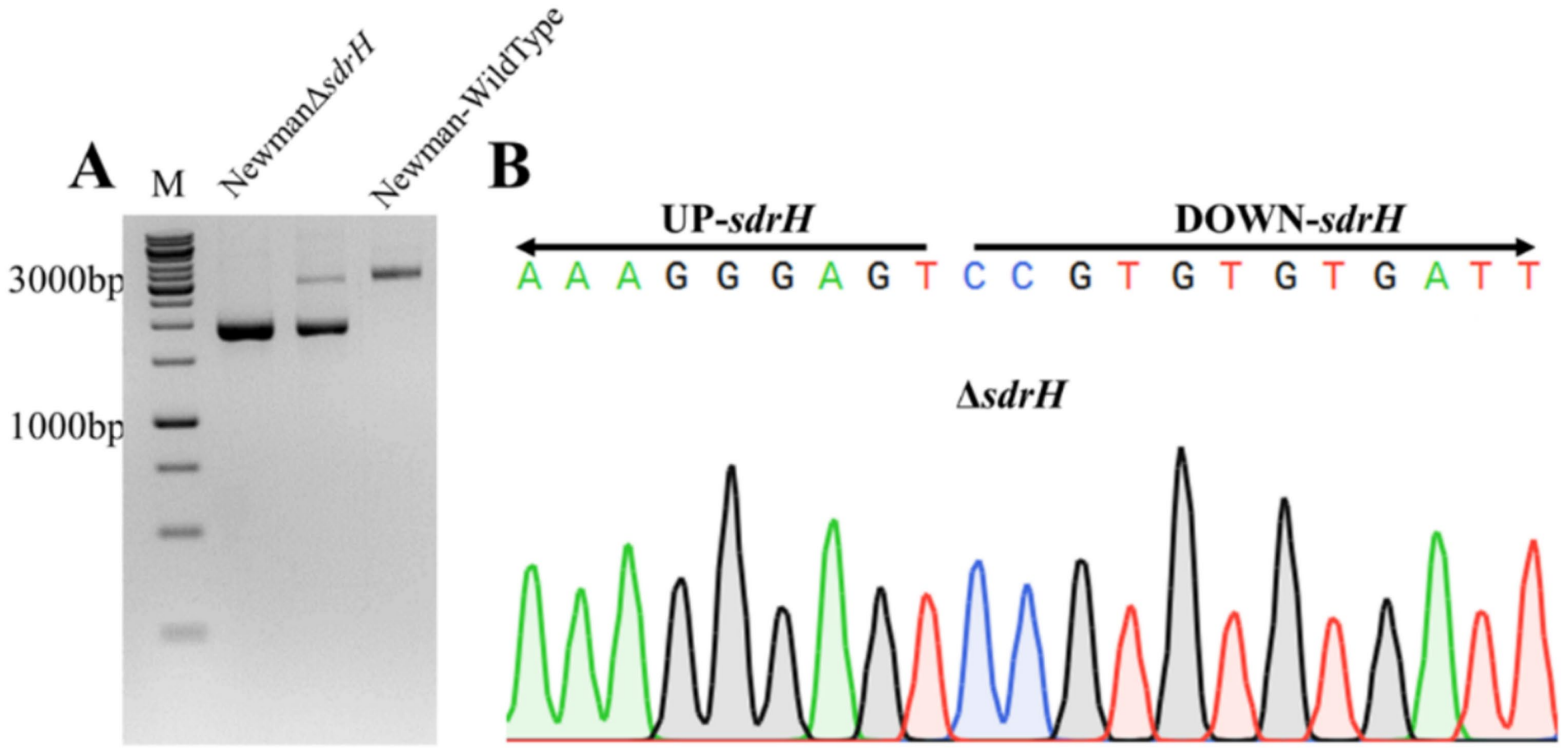
**(A)** Electrophoresis of DNA from the *Staphylococcus aureus* Newman wild-type strain and the suspected icaA-C mutant strain; **(B)** Sequencing to verify the successful mutation of the NewmanΔsdrH strain.

**Figure 4:**
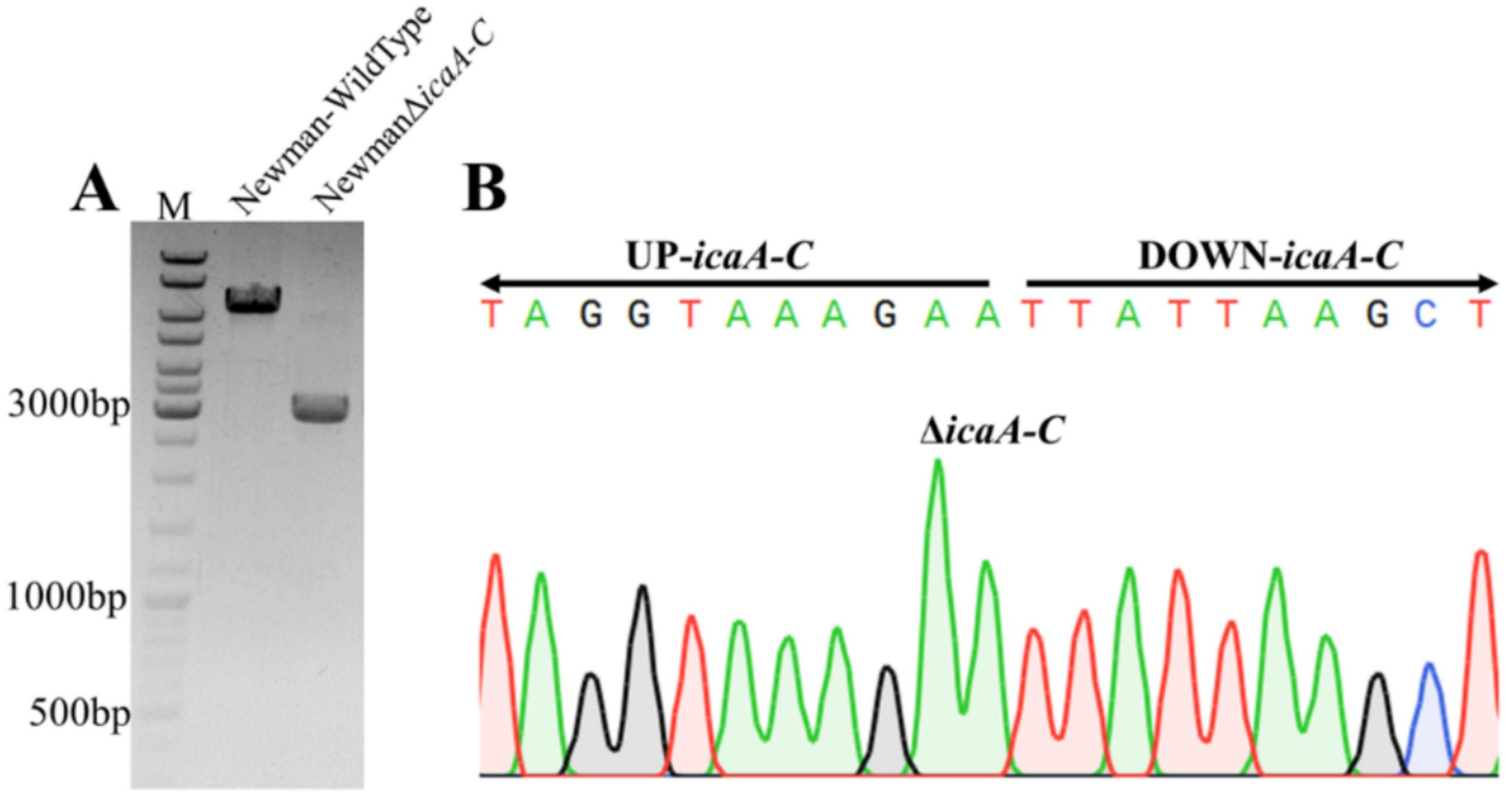
The electrophoretic profiles of **(A)** the *Staphylococcus aureus* Newman wild-type strain and the suspected icaA-C mutant; **(B)** the NewmanΔicaA-C strain demonstrating a successful mutation, as confirmed by sequencing.

To further investigate the roles of *sdrH* and *icaA*-*C* in *S. aureus*-caused DFUs, the related gene mutants were generated with homologous recombination strategy. Successfully, the *sdrH* and *icaA-C* single mutant as well as the sdrH/icaA-C double mutant strain were constructed (Figures 3A,B, 4A,B).

### 2.3 Sdrh promotes biofilm formation in *Staphylococcus aureus*

**Figure 5:**
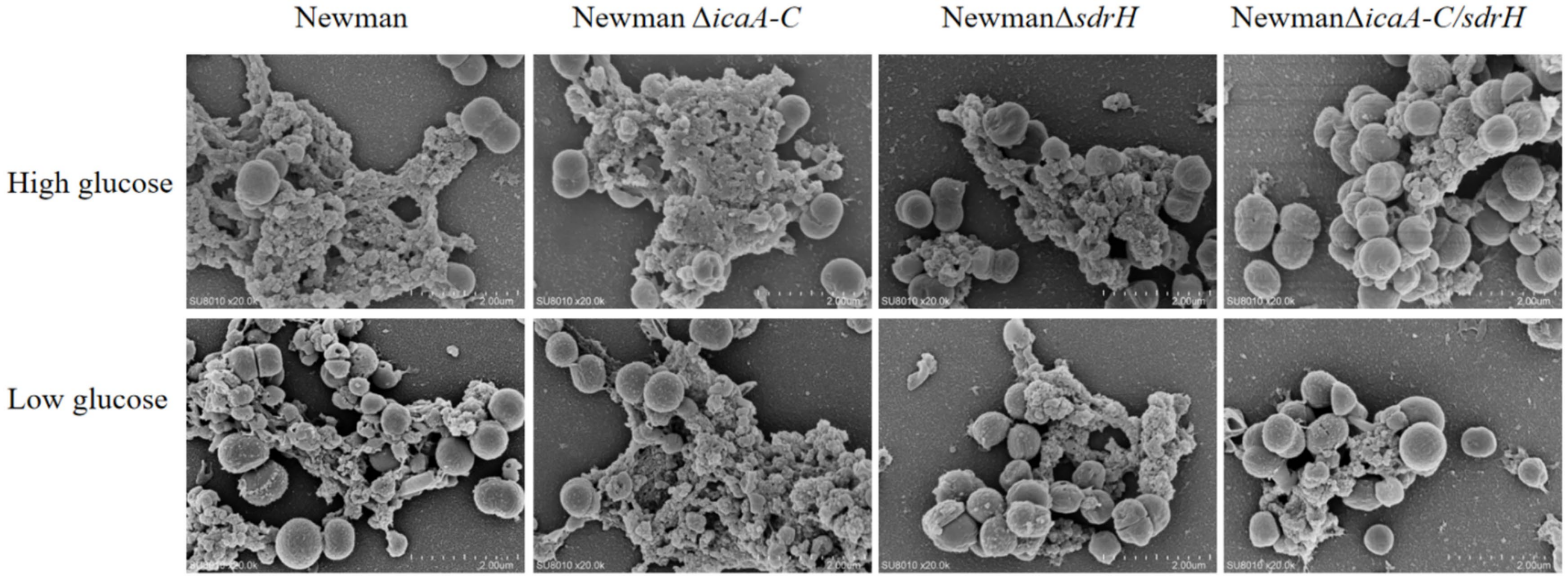
Biofilm formation of four different strains of *S. aureus* in high and low sugar DMEM media, respectively.

Aureobasidium spp. were cultured into high and low sugar DMEM medium, and biofilm formation was measured after 48 h of incubation. By scanning electron microscopy, we found that the presence of the sdrH gene in the high sugar state could help *S. aureus* to form more biofilms during the proliferation process (Figure 5). While comparing the biofilm expression of the four *S. aureus* different strains in their low sugar condition, we found that the presence of the sdrH gene could help *S. aureus* form more biofilms, but there was a gap compared to the amount of sdrH gene promoting the biofilm expression of *S. aureus* in the high sugar culture condition. In contrast, the icaA-C gene mutation had no significant effect on biofilm formation in *S. aureus* regardless of the environment. Therefore, we conclude that *S. aureus* can form biofilms through the expression of the sdrH gene, and that glucose in a high sugar environment promotes the up-regulation of the expression of the sdrH gene, assisting in the production of more biofilms and enhancing its colonisation ability.

### 2.4 *sdrH* enhances *Staphylococcus aureus* infection in diabetic wounds

**Figure 6:**
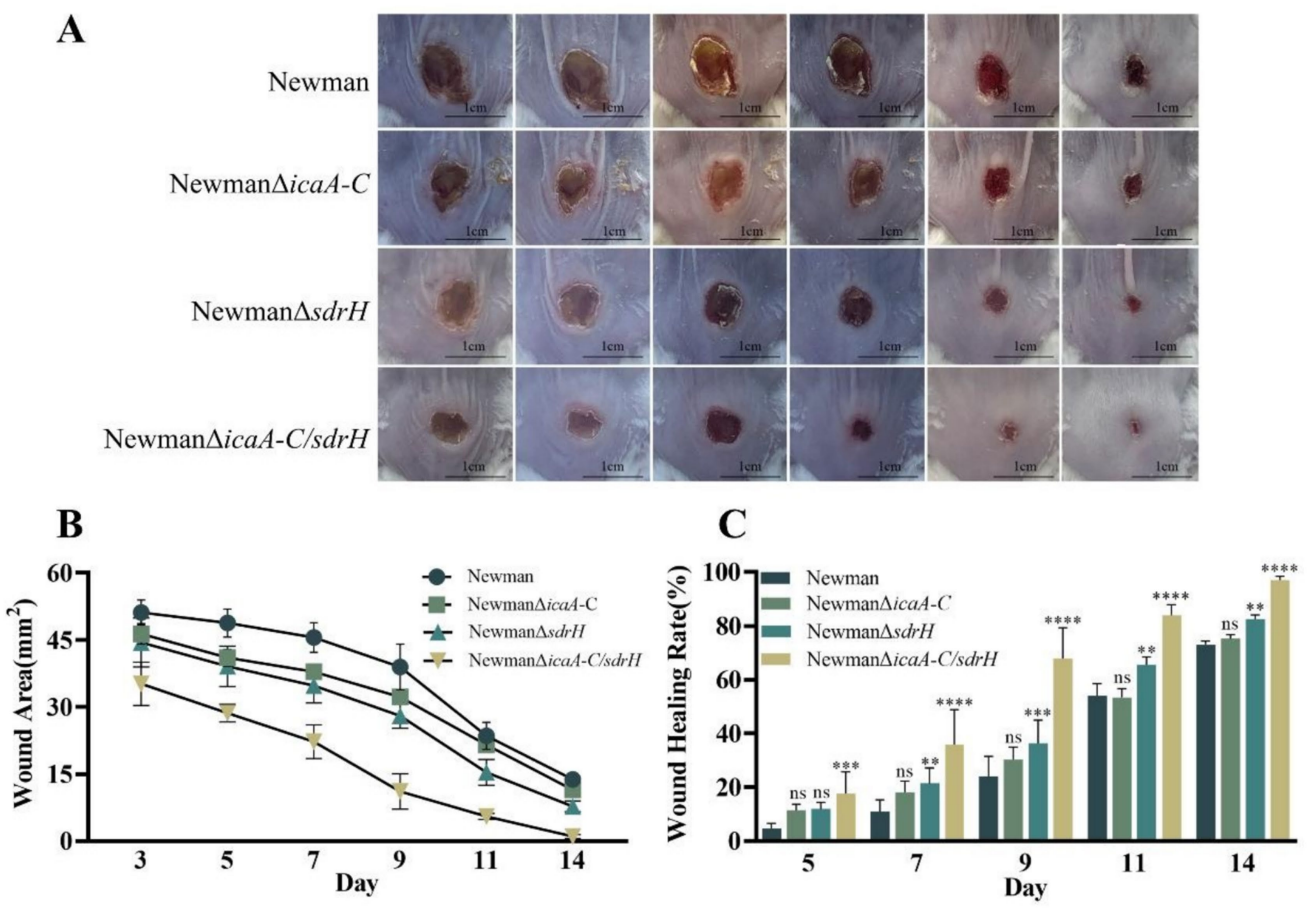
**(A)** Documentation of wound progression at 14 days for the diabetic mice infected with the four experimental strains. **(B)** Statistical analysis of the mean wound area at 14 days for diabetic mice infected with the four experimental strains. **(C)** The rate of wound healing over the 14-day period for the diabetic mice infected with the four experimental strains. Statistical significance was determined using a two-way analysis of variance (2-way ANOVA), with * indicating *p* < 0.05, ** indicating *p* < 0.01, *** indicating *p* < 0.001, **** indicating *p* < 0.0001, and ns indicating non-significance.

**Figure 7:**
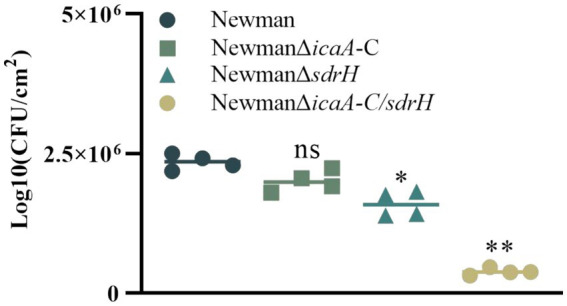
On day 5, the bacterial colonization of diabetic wounds infected by four strains was assessed. One-way analysis of variance (ANOVA) was employed to ascertain statistical significance, with * indicating *p* values < 0.05, ** indicating p values < 0.01, and ns indicating non-significance.

The *sdrH* gene encodes the serine-aspartic repeat protein H (SdrH), which belongs to a family of staphylococcal adhesion proteins containing a serine-aspartic repeat (SDR) domain and is essential for *Staphylococcus aureus* infection (Kim et al., 2021). The function of *S. aureus* adhesins in DFUs remains elusive. Experimental outcomes indicated that the NewmanΔ*sdrH* mutant exhibited substantial attenuation of pathogenicity relative to the wild-type strain, as evidenced by the accelerated healing of back wounds in diabetic mice (Figures 6A,B). The result also demonstrated the markedly enhanced rate of wound closure in these mice (Figure 6C). The SdrH significantly augmented the colonization of *S. aureus* in the wounds (Figure 7). Overall, throughout the infection process caused by *Staphylococcus aureus*, the sdrH gene enhances the bacteria’s adhesion capabilities, resulting in a significant increase in bacterial colonization within the infected wound. This escalation in colonization subsequently affects the wound’s healing process.

### 2.5 *icaA-C* and *sdrH* together promote *Staphylococcus aureus* bacterial colonization and delay wound healing

Expression of the icaA-C gene cluster leads to the production of a polysaccharide intercellular adhesin (PIA)-dependent biofilm (Cramton et al., 1999), which enhances the colonization capabilities of *S. aureus*. Our results showed that there was no significant difference in wound size between mice infected with the NewmanΔ*icaA-C* and the wild-type strain. To ascertain whether the concurrent elimination of icaA-C and sdrH would reduce the infective capacity of *Staphylococcus aureus*, a double mutant NewmanΔicaA-C/ΔsdrH was generated. Remarkably, a reduction in the wound healing duration in mice infected with NewmanΔicaA-C/ΔsdrH was observed (Figure 6). Furthermore, the bacterial burden in the wounds was substantially reduced compared to that observed in the Newman- and NewmanΔ*sdrH*-infected cohorts, as shown in Figure 7. The findings showed that the simultaneous knockout of the sdrH and icaA-C genes can mitigate the colonization of *S. aureus* within wounds, diminish wound infections, enhance the host’s repair capacity, and facilitate wound healing.

### 2.6 *icaA-C* synergizes with *sdrH* to reduce the host immune response to clear *Staphylococcus aureus*

**Figure 8:**
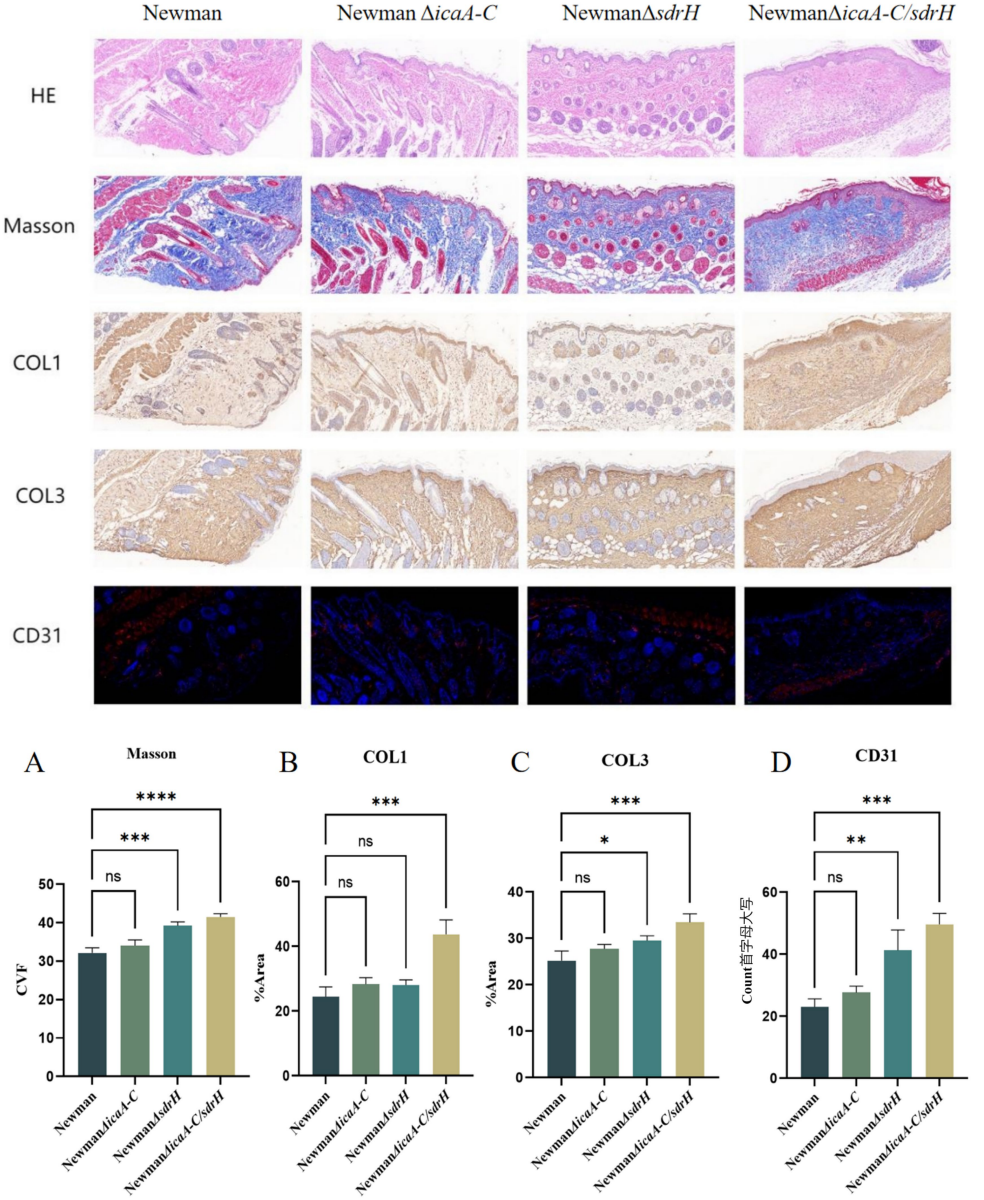
The statistical analysis encompasses the outcomes of diabetic wounds derived from four distinct groups of strains. **(A)** The collagen volume fraction as determined by Masson’s trichrome staining after a 5-day interval. **(B)** The collagen I statistics of the diabetic wounds from the four strain groups at the same 5-day mark. The collagen 3 statistics **(C)** and the vessel number statistics **(D)** of diabetic wounds from four groups of strains after a 5-day incubation period. Statistical significance was determined utilizing One-Way ANOVA, with * indicating *p* < 0.05, ** indicating *p* < 0.01, *** indicating *p* < 0.001, **** indicating *p* < 0.0001, and “ns” denoting non-significance.

The histopathological examination of mouse back wounds on day 5 post-infection was performed. Masson’s trichrome staining results revealed that the protein sdrH mitigated the production of type I and type III collagen fibers, abrogated the differentiation of fibroblasts, and perturbed the healing and regenerative processes of host wounds. Moreover, immunofluorescence assay results indicated that SdrH influences angiogenesis during wound mending and re-epithelialization, which in turn delays wound closure by impeding the recovery of vascular networks. Additionally, greater COL1 and COL3 collagen fiber production was observed, and enhanced neovascularization was evident around the wound site (Figure 8). These findings indicate that icaA-C may aid sdrH in modulating the host’s immune response during *Staphylococcus aureus* infection of wounds, which in turn promotes the pathogen’s ability to colonize the wound and slows wound healing. Collectively, the deletion of the sdrh gene may affect the expression of specific pathways, reduce the colonization ability and virulence of *Staphylococcus aureus* in wounds, and enhance the immune system’s capacity to eliminate the bacteria. This alteration can subsequently modify the intracellular environment of the host wound, facilitating the regeneration of wound collagen and microvasculature, thereby accelerating the healing of the host wound surface.

## 3 Discussion

Diabetes mellitus, a chronic condition, is characterized by hyperglycemia, which leads to a range of systemic manifestations and diverse local pathological responses within the wound environment. These responses include chronic inflammation, dysregulated angiogenesis, hypoxia-induced oxidative stress, neuropathy, the formation of advanced glycation end products, and the disruption of neuropeptide signaling (Baltzis et al., 2014). *Staphylococcus aureus* is the predominant gram-positive opportunistic pathogen implicated in skin infections worldwide (Wong et al., 2015). Infections caused by *Staphylococcus aureus* infection have the potential to significantly delay the wound healing. This bacterium predominantly secretes three principal virulence factors, which play critical roles throughout the various stages of infection. Our investigation revealed that sdrH is a determinant in the prolonged healing of diabetic wounds following *Staphylococcus aureus* infection, with the *icaA*-*C* factors exacerbating this effect (Figure 5C). SdrH is a protein with a serine–aspartate dipeptide repeat sequence. Research conducted by Mccrea et al. (2000) has indicated that SdrH may play a role in the pathogenesis of *Staphylococcus epidermidis* infections. Moreover, studies by Muthukrishnan et al. (2011) suggests a potential association between SdrH and the colonization of the nasal mucosa by *Staphylococcus aureus*. This study indicates that sdrH may play a role in the colonization of diabetic wounds by *Staphylococcus aureus*. As a crucial adhesin of *Staphylococcus aureus*, SdrH promotes bacterial adhesion within the wound during the colonization phase, thereby enhancing infection by facilitating bacterial establishment. Additionally, the polysaccharide intercellular adhesin PIA, encoded by the ica operon, is deemed a critical factor in the formation of staphylococcal biofilms (Kot et al., 2020). Intercellular adhesive sites, encoded by the four genes within the operon (icaADBC), are vital for the production of PIA (Arciola et al., 2015). The biofilm formed by *Staphylococcus aureus* plays a crucial role in evading host immune system-mediated clearance mechanisms during bacterial colonization of wound sites. The gene products of the ica cluster are essential for the assembly and pathogenicity of *Staphylococcus aureus* biofilms, and their expression is significantly upregulated under anaerobic growth conditions (Cramton et al., 1999). In this study, the knockout of the icaA-C genes in *Staphylococcus aureus* ddid not exhibit any significant impact on the pathogenesis of diabetic wounds, as depicted in Figure 5. These findings indicate that the *ica* locus has a minimal impact on the infection process. Additionally, research by Archer et al. demonstrated that although the *ica* locus is crucial for biofilm formation, biofilm production can occur independently of ICA, and PIA is not a necessary element for the initiation of biofilm assembly (Archer et al., 2011). Consequently, in the current investigation, the deletion of the *ica* gene cluster in *Staphylococcus aureus* appears to have prompted the utilization of alternative biofilm formation mechanisms; nevertheless, the colonization capacity of *Staphylococcus aureus* remained unaffected. Importantly, the concurrent deletion of the *icaA*-*C* and sdrH genes resulted in a significant reduction in the ability of the NewmanΔ*icaA*-*C*/ΔsdrH strain to infect wounds and establish colonization, compared to the NewmanΔsdrH strain (Figures 5, 6). Upon infection with the NewmanΔ*icaA-C/*Δ*sdrH* strain, the host immune response was significantly enhanced, as evidenced by augmented inflammatory cell infiltration, elevated production of collagen types COL1 and COL3, and increased angiogenesis (Figure 7). These findings indicate that *icaA*-*C* and sdrH synergistically enhance the infectivity of *Staphylococcus aureus* during diabetic wound infections; however, the specific mechanisms underlying their activities remain to be elucidated.

## 4 Materials and methods

### 4.1 Strain and culture

In the present study, the primary objective was to isolate the Newman strain of *Staphylococcus aureus* (NCTC 8178, ST1/agr I) from throat swab samples obtained from patients experiencing secondary infections associated with tuberculous osteomyelitis (Duthie and Lorenz, 1952; Missiakas and Schneewind, 2013). The culture was subsequently inoculated and incubated at 37°C following the reanimation of *Staphylococcus aureus*, which had been preserved at −80°C, on Trypticase Soy Broth (TSB) agar until distinct bacterial colonies formed. Thereafter, the selected bacterial colonies were cultured overnight under conditions of agitation at 200 rpm.

### 4.2 Construction of a mouse model of DU and *Staphylococcus aureus* infection

Female BALB/c mice, aged 6 weeks, were procured from the Experimental Animal Center of Army Medical University (AMU). The study was conducted in adherence with the guidelines outlined in the Regulations on the Administration of Experimental Animals, as ratified by the State Council of the People’s Republic of China. Following 1 week of adaptive feeding, the mice were progressively transitioned to a diet high in fat and sugar content over 30 days, during which their weight fluctuations were meticulously monitored. Streptozotocin (STZ) was subsequently administered at a dosage of 60 mg per kilogram of body weight over a span of five consecutive days to establish a diabetic mouse model. Prior to each injection, the mice were fasted for a 12-h period. Plasma glucose levels were assessed on the 7th day post-injection, and a blood glucose level ≥16.7 mmol/L was deemed indicative of the establishment of a diabetic mouse model (Vågesjö et al., 2018). The dorsal regions of each mouse (both the control and diabetic mice) were subsequently shaved, and a full-thickness skin lesion of approximately 10 mm in diameter was created. This wound was subsequently sutured with a rubber ring to prevent inherent skin contraction (Michaels et al., 2007). A suspension of *Staphylococcus aureus* Newman, containing 1 × 10^7^ colony-forming units (CFUs), was instilled into the skin wounds of both diabetic and control mice.

### 4.3 Identification of virulence genes and establishment of knockout mutants

On the fifth day post-infection, both diabetic and control mice were euthanized. The wound tissue and pus collected from the mice were processed using the TRIPURE method. Following homogenization and grinding of the samples, bacterial RNA was extracted using chloroform and isopropyl alcohol. The precipitate was analyzed to confirm that the OD260/280 and OD260/230 ratios exceeded 1.8. After eliminating genomic contamination from the validated RNA, cDNA reverse transcription was performed using the Invitrogen SuperScript IV cDNA synthesis kit. To replicate the host body’s growth conditions for *S. aureus*, the bacteria were concurrently introduced into standard DMEM and high glucose DMEM (4.5 g/L). RNA was then extracted from bacterial cultures during the logarithmic growth phase using an RNA extraction kit. The obtained RNA was utilized with the RevertAid First Strand cDNA Synthesis Kit from ThermoFisher. Specifically, 5 μg of RNA was taken and mixed with 1 μL of Random Hexamer primer, followed by the addition of ribozyme-free water to a final volume of 12 μL. After incubating at 65°C for 5 min, 4 μL of 5X Reaction Buffer was added, along with 1 μL of RiboLock RNase Inhibitor (20 U/μL), 2 μL of 10 mM dNTP Mix, and 1 μL of RevertAid M-MuLV RT (200 U/μL), bringing the total volume to 20 μL. The mixture was thoroughly mixed and incubated at 25°C for 5 min, followed by an incubation at 42°C for 60 min. After this period, the transcribed cDNA can be obtained. The concentration of the resulting cDNA was quantified with a spectrophotometer, diluted with DEPC water to achieve a uniform concentration, and utilized as a template to analyze the expression levels of forty genes associated with adhesins, toxins, and immune evasion. Primers can be found in the Supplementary materials. Primers can be found in the Supplementary materials. To create a *Staphylococcus aureus* Newman strain with gene knockouts of the upregulated genes, the temperature-sensitive *E. coli*-*Staphylococcus aureus* shuttle plasmid pBT2 was employed based on primer design surrounding the target gene (Brückner, 1997). Each resulting mutant underwent thorough verification through PCR amplification and subsequent sequencing. The sdrH gene knockout strain was designated as NewmanΔsdrH. In conclusion, this study successfully constructed the icaA-C gene cluster knockout strain NewmanΔicaA-C and the icaA-C/sdrH double knockout strain NewmanΔicaA-C/ΔsdrH.

### 4.4 Real-time reverse transcription-PCR

RT-qPCR detection was conducted using the BioRad Sso Advanced Universal SYBR® Green master mix. The obtained cDNA was diluted 100-fold with DEPC-treated water and utilized as a template. The SYBR® Green qPCR mixture was prepared, and three biological replicates were performed, with each sample set up in triplicate wells. For each reaction, 10 *μ*L of 2 × Sso Advanced SYBR® Green master mix, 1 μL of each upstream and downstream primer, 1 μL of the diluted template, and 7 μ L of DEPC-treated water were combined. Use the CFX96 real-time fluorescence quantitative PCR instrument (Bio-Rad of the United States, 1,855,201). The thermal cycling conditions included pre-denaturation at 95 ° C for 3 min, denaturation at 95 ° C for 10 s, annealing at 60°C for 10 s, and extension at 72°C for 30 s, with a total of 42 cycles. The dissociation curve was generated by increasing the temperature from 58°C to 95°C in increments of 0.5°C, maintaining each increment for 5 s to measure changes in fluorescence intensity. A single peak in the dissociation curve at the annealing temperature indicates high specificity.

### 4.5 Biofilm production by *Staphylococcus aureus*

Four different strains of *S. aureus* were inoculated into Brain Heart Infusion (BHI) slant medium respectively, and cultured for 18–24 h, then washed down the bacterial foetus on the slant to form bacterial suspension with PBS (phosphate buffer solution), washed with PBS for 3 times, and then the 96-well plate was 100ul per well, with 3 replicate wells for each bacterium, and its OD value was measured in the enzyme marker at 570 nm, and then the bacterial suspension was configured as 0.5OD with DMEM The medium (high sugar and low sugar) was configured as 0.5OD bacterial suspension. 24-well plates were placed in sterile slides and 0.5 mL of 0.5OD bacterial suspension was added to each well, and the biofilm was prepared by incubation for 6 days at 37°C. The medium in the wells was washed out after 6 days, and washed with PBS for 2–3 times, and then 2.5% glutaraldehyde was added to each well for pre-fixation, and then 1% osmium tetroxide for post-fixation was added to the wells. After washing the fixative in PBS solution at pH 7.4, it was fully dehydrated by graded ethanol (30, 50, 70, 90, 100% ethanol twice) and finally critical point dried and coated for observation. Using Hitachi SU-8010 field emission scanning electron microscope to observe the biofilm and take screenshots for subsequent statistics.

### 4.6 Wound healing in a mouse model of knockout *Staphylococcus aureus* strain-infected DU

Following the establishment of the mouse model of DU, the experimental cohort of diabetic mice, consisting of 10 individuals, was inoculated with a gene knockout strain at a density of 1 × 10^7^ CFU. Concurrently, 10 control mice were infected with the Newman strain at the same dose, and subsequent colonization by the wild-type strains was permitted. Continuous monitoring of wound closure was conducted, with images being captured at 3, 5, 7, 9, 11, and 14 days post-bacterial colonization to determine the wound healing velocity. Tissue samples from the wounds were collected on days 1, 3, 7, and 14. They were subsequently fixed in a 4% tissue fixative solution for 48 h, followed by paraffin embedding and staining with hematoxylin–eosin (HE) and Masson’s trichrome. Immunofluorescence was used to detect the expression of CD31 (with an anti-CD31 mouse monoclonal antibody), while immunohistochemistry was used to detect collagen expression (with a mouse anti-collagen I antibody and an anti-collagen type III polyclonal antibody). Immunofluorescence was employed to assess vascular formation through ImageJ analysis. The image to be analyzed is opened in ImageJ, converted to 8-bit format, and bright field images are processed to identify branch points and intersection points for statistical analysis. A similar approach is utilized for immunohistochemistry. The results of immunohistochemistry are analyzed using ImageJ to quantify collagen area and vascular formation. In this process, the image is opened in ImageJ, and image deconvolution is applied to separate the DAB and hematoxylin channels. The DAB color development signal window is selected, and the grayscale signal is converted to optical density values. The upper and lower limits of the threshold are manually adjusted to encompass the ideal area, allowing for quantitative data analysis.

### 4.7 Statistical analysis

All experiments were conducted a minimum of three times in triplicate, with each sample representing a collective from at least three distinct culture dishes or three individual samples. Statistical analyses were performed via GraphPad Prism version 9.3 (GraphPad Software, Inc., USA). An unpaired t test was used to compare the mean values between the two groups. Furthermore, two-way ANOVA was utilized to assess the differences in cytokine levels and wound healing rates, whereas one-way ANOVA was adopted for the comparison of skin wound bacterial loads. The results are presented as the means ± standard deviations (SDs), with a significance level of *p* < 0.05.

## 5 Conclusion

The immune response serves as a critical defense mechanism for the host to actively combat infections caused by *Staphylococcus aureus*. The sdrH and icaA-C genes, which encode *Staphylococcus aureus* adhesins, play pivotal roles in the infection process, specifically in the context of diabetic wounds. Upon the deletion of sdrH, the pathogenic potential of *Staphylococcus aureus* was attenuated, leading to a reduction in bacterial colonization of the wound site and acceleration of the wound healing process. Similarly, disruption of the icaA-C gene cluster mitigated infection. Our findings indicate that sdrH and icaA-C are pivotal in the progression of *Staphylococcus aureus* infection within diabetic wounds. The development of pharmacological agents capable of suppressing the expression of sdrH and icaA-C may significantly benefit the management of diabetic wound infections.

## Data Availability

The original contributions presented in the study are included in the article/Supplementary material, further inquiries can be directed to the corresponding authors.
