# Supplementary material for: sdrH enhances Staphylococcus aureus infection in diabetic wounds
Source: Front Microbiol. 2025 Jun 19;16:1502428. doi: 10.3389/fmicb.2025.1502428 (PMC12222116; doi:10.3389/fmicb.2025.1502428)
Supplement: Supplementary file 1 [file Table_1.docx]

Table1 Knockout primer sequence.

| Gene | **Sequences**（5’-3’） |
| --- | --- |
| pBT2 | F ccgggtaccgagctcg |
|  | R taatgcggtagtttatcacagtt |
| NM-icaADC | F tcgagctcggtacccggggatccaataccttgcgtaacccgt |
|  | F2 gcgtgtttttaacatagcttaataattctttacctacctttcgttagtta |
|  | R1 taactaacgaaaggtaggtaaagaattattaagctatgttaaaaacacgc |
|  | R2 gataaactaccgcattaaagcttgcggtcgtgtagattatggt |
| NM-icaADC-test | F taactaacgaaaggtaggtaaaga |
|  | R tcccattggcatttacga |
| NM-sdrH | F1 tcgagctcggtacccggggatccctttatcatttctgtcccattc |
|  | F2 aaaacgaatcacacacggactccctttttcatgtaagtattaa |
|  | R1 ttaatacttacatgaaaaagggagtccgtgtgtgattcgtttt |
|  | R2 gataaactaccgcattaaagctttgggcatcattgctaact |
| NM-sdrH-test | F ttggggtgggctctaaca |
|  | R agcatgattgttcctcctta |

Tabel2 Amplification primer sequence.

| Gene | **Sequences**（5’-3’） |
| --- | --- |
| *16s* RNA | F tgagatgttgggttaagtcccgca |
|  | R cggtttcgctgccctttgtattgt |
| *adsa* | F cgactagccgaagaaaaaggg |
|  | R taccgactgccatagcatcataa |
| *aur* | F gatggtgttcatgcacctgaca |
|  | R attcgttggctttactttcttcg |
| *clfA* | F tctggtacgactgtgtatccgc |
|  | R tggtggcactttagcagttgaa |
| *clfB* | F ttacagaccgagcaaaggcac |
|  | R tgcttgtatgtgttttgacctgaa |
| *eap* | F ttagcatcaacaggtgcaaact |
|  | R tgattgcatatggaacatggact |
| *ecb* | F acgtttgccggtgaatctcat |
|  | R cagctctttgtgctttacggtgt |
| *efb* | F gcacgtccacaatttaataaacca |
|  | R tcaattcgctcttgtaagaccatt |
| *emp* | F gaaatcaataatcgcgtgaatgtag |
|  | R gtttgcgtagtaatgaagtggtgg |
| *essA* | F ggcaaggttcagaccaaatcc |
|  | R acggcatcagcagtgctattc |
| *essB* | F gatcaagcgaaacaattagcgg |
|  | R caccttattggcgaactgtcctt |
| *fnbA* | F caatgatcgttgttgggatgg |
|  | R tgcgtttgacggttgttctgt |
| *fnbB* | F actgaaagtaaagcaagcgaaaca |
|  | R tccgatggcaaatcaactcg |
| *hla* | F gtggtttagcctggccttca |
|  | R cgaaacatttgcaccaataagg |
| *hlb* | F ggtgggacaaaactgaaggtagc |
|  | R tgctatcattatcgaatccacaacc |
| *hlgB* | F atgttggctggggagttgaa |
|  | R gcgtatgcactgctttgtctgc |
| *hrtA* | F atggtgcctctggttctggg |
|  | R cgtaaatcactaggacgatgctgtt |
| *hrtB* | F attgtgctattgaacgataacggat |
|  | R tgcttgctctgcttgataactcg |
| *icaA* | F tcgcactctttattgatagtcgct |
|  | R gtattccctctgtctgggcttg |
| *icaC* | F aggtcaatggtatggctattttatcg |
|  | R acggtatcgtgaaacgctgtg |
| *icaD* | F atacccaacgctaaaatcatcgc |
|  | R gcaacacgtattgtattgatactttcg |
| *isdA* | F cgcagacagccaacaagtca |
|  | R tgaagagccatctttttgcact |
| *isdB* | F ttgaggcccctacttctgaaac |
|  | R ttggacgagagtttggtgcg |
| *isdC* | F ttctgcttcaggtagtgacaaagg |
|  | R tgcgattaataatgctaaggatgc |
| *isdH* | F ctaggcgttgcatcggtca |
|  | R gcaggatagtttttcgcagtgtt |
| *lgt* | F aggtggctttattgctggtgtt |
|  | R ctcgtgattcataaagttacccca |
| *mntC* | F caaagcagtgataagtcaaatggc |
|  | R cgttgtataaaataacgtcagcgtc |
| *nuc* | F ggtgaaaccgaatacgcctgta |
|  | R ctctagcaagtcccttttccactaa |
| *pvlF* | F acacctaaagacaaaatgcctgtaac |
|  | R agaccaatagccccagaaacca |
| *sak* | F gaatgggcattagatgcgaca |
|  | R tgctctgataaatctgggacaaca |
| *sasF* | F cgaaattaggaaaagctgaagca |
|  | R aagacgtgtcgccagttgatg |
| *sbi* | F ggggaagcaaaagcgagtg |
|  | R tgcacgttctgggtgttcg |
| *scinB* | F aaacgcatagcagaagaattaagaac |
|  | R tgcaactttagcatcagccatt |
| *sdrC* | F aatgaaaggccaaacaagcag |
|  | R gttgatgagccattcacatttga |
| *sdrH* | F cgctcatttgaaccgcatg |
|  | R tcgtcgctgtgattcgttttta |
| *spa* | F gccaaagtgctaacctattgtcag |
|  | R gggtcatcttttaggctttgga |
